# Aquatic insects differentially affect lake sturgeon larval phenotypes and egg surface microbial communities

**DOI:** 10.1371/journal.pone.0277336

**Published:** 2022-11-21

**Authors:** Ryan W. Walquist, Kim T. Scribner, Justin Waraniak, John M. Bauman, Terence L. Marsh, Jeannette Kanefsky, Douglas L. Larson

**Affiliations:** 1 Department of Fisheries and Wildlife, Michigan State University, East Lansing, Michigan, United States of America; 2 Department of Integrative Biology, Michigan State University, East Lansing, Michigan, United States of America; 3 Michigan Department of Natural Resources, Gladstone, MI, United States of America; 4 Department of Microbiology and Molecular Genetics, Michigan State University, East Lansing, Michigan, United States of America; Central University of South Bihar, INDIA

## Abstract

Documentation of how interactions among members of different stream communities [e.g., microbial communities and aquatic insect taxa exhibiting different feeding strategies (FS)] collectively influence the growth, survival, and recruitment of stream fishes is limited. Considerable spatial overlap exists between early life stages of stream fishes, including species of conservation concern like lake sturgeon (*Acipenser fulvescens*), and aquatic insects and microbial taxa that abundantly occupy substrates on which spawning occurs. Habitat overlap suggests that species interactions across trophic levels may be common, but outcomes of these interactions are poorly understood. We conducted an experiment where lake sturgeon eggs were fertilized and incubated in the presence of individuals from one of four aquatic insect FS taxa including predators, facultative and obligate-scrapers, collector-filterers/facultative predators, and a control (no insects). We quantified and compared the effects of different insect taxa on the taxonomic composition and relative abundance of egg surface bacterial and lower eukaryotic communities, egg size, incubation time to hatch, free embryo body size (total length) at hatch, yolk-sac area, (a measure of resource utilization), and percent survival to hatch. Mean egg size varied significantly among insect treatments. Eggs exposed to predators had a lower mean percent survival to hatch. Eggs exposed to predators had significantly shorter incubation periods. At hatch, free embryos exposed to predators had significantly smaller yolk sacs and total length. Multivariate analyses revealed that egg bacterial and lower eukaryotic surface community composition varied significantly among insect treatments and between time periods (1 vs 4 days post-fertilization). Quantitative PCR documented significant differences in bacterial 16S copy number, and thus abundance on egg surfaces varied across insect treatments. Results indicate that lethal and non-lethal effects associated with interactions between lake sturgeon eggs and free embryos and aquatic insects, particularly predators, contributed to lake sturgeon trait variability that may affect population levels of recruitment.

## Introduction

Predicting changes in species and population trait distributions, species abundance, or community composition necessitates greater understanding of the outcomes of species interactions [1, 2], particularly in situations involving low abundance species of conservation concern [3]. Stream environments are characterized by many abiotic and biotic factors that can affect species interactions, including species development, behavior, and survival [4–6]. The degree of species interactions should increase as habitat overlap increases [7], especially when habitats are confined by narrow boundaries (e.g., stream banks), and when species obligately and concurrently occupy the same habitats.

Erosional stream regions selected as preferred spawning habitats by many fish species typically are characterized by flowing water and abundant hard substrata with interstitial spaces that provide refuge for fish eggs and early post-hatch life stages. Interstitial habitats also support diverse benthic communities [8, 9]. Benthic insects and early life stages of lithopelagophilic fish (e.g., eggs and free embryos) occupy the interstitial spaces of river substrate [10]. The degree of habitat overlap suggests interactions between aquatic insects and fish eggs and free-embryos are common but of unknown effect.

Physical and biotic environmental conditions obligately utilized by females of many fish species for spawning can significantly influence offspring phenotypic traits [11], and can affect embryonic and larval developmental time [12, 13] that are associated with survival [14]. Aquatic insects are characterized by adaptations reflecting long-standing associations with predators and prey [15]. The effects of aquatic insects on fish eggs and free-embryos may be inferred from the feeding behavior associated with different aquatic insect groups [16]. Different groups of aquatic insects, often representing taxa from several orders, frequently share behavioral and/or morphological adaptations used to acquire resources [16, 17]. For example, adaptations include scraping mandibles present in scraping insects that are used to remove microbial biofilm from substrate surfaces [18], and setae clusters characteristic of some collector-filterer insects which are used to capture particulate debris [19].

It is important to consider not only the lethal effects of aquatic insects [15, 20], and microbes [21, 22] on fishes during vulnerable early life stages, but also the non-lethal effects including behavioral and physiological phenotypic responses [23, 24], trait-mediated indirect interactions [25], and the timing of ontogenetic shifts such as hatching and absorption of endogenous yolk reserves [22]. Life history plasticity such as hatching and pre-feeding resource use in response to predatory cues is a common phenomenon [26]. Predators that induce early hatching in fish may affect the quality (i.e., body size and amount of yolk-sac endogenous reserves) of free embryos at hatch, indirectly affecting susceptibility to predators during subsequent life stages [27]. Eggs from non-fish taxa have been shown to reduce incubation periods in response to cues from predatory insects [28]. Some fishes have demonstrated plasticity in hatch time in response to pathogens, and to chemical cues produced by injury or predation on conspecifics [27, 29, 30], but the mechanism is not well understood [31].

Oviparous fishes spend their entire life from fertilization through death in a dilute solution of microbes that include bacteria, Archaea, viruses and lower eukaryotes (e.g., fungi and oomycetes; [32]). Eggs extruded into the water column during spawning become a substrate to which bacteria and lower eukaryotes adhere, subsist, and flourish. Because bacteria, lower eukaryotes, and insects are ubiquitously distributed in aquatic environments [33], including biofilms distributed over hard stream substrates [34, 35], greater focus would be beneficial to foster understanding of interactions across trophic levels, with specific emphasis on ‘bottom-up’ interactions involving microbial taxa (bacteria and lower eukaryotes including fungi and oomycetes). Research has shown that some interactions between microbes and hosts are both deleterious (e.g., promotes embryo developmental arrest during incubation) and advantageous (probiotic via negative interactions with other pathogenic microbes) to fish eggs and developing embryos [22, 33, 36, 37].

Lake sturgeon (*Acipenser fulvescens*) are a highly fecund [38] adfluvial lithopelagophilic fish that spawns on hard (rock and gravel) substrate [39] in fast-flowing regions of rivers. Due to the aggregated distribution and limited mobility of eggs and newly hatched free embryos, individuals in these early life stages are highly vulnerability to predation [40]. A spawning population of lake sturgeon exists in the Upper Black River in the northern lower peninsula of Michigan where well-defined spawning areas have been documented [41]. These spawning areas are composed of interspaced riffle-pool-run stretches with substrata composed of course cobble that include >38 families of benthic insects [42]. A variety of insect taxa characterized by different feeding strategies (FS) are commonly found in the spawning areas, including predators, scrapers, and collector-filterer taxa [43, 44] that are both food and predators of other stream community members.

Empirical data are needed that can evaluate the relative lethal and non-lethal effects of multiple trophic levels in stream communities on fish phenotypic and behavioral trait variation across ontogenetic stages [45]. Understanding how and under what circumstances fish, aquatic insects, and microbes interact positively or negatively under future environmentally-induced changes in freshwater aquatic ecosystems is essential to ecosystem sustainability [46], and to the ability of aquatic ecosystems to provide essential ecosystem services [32]. Maintenance of fish populations requires greater understanding of relationships between recruitment and environmental characteristics including stream hydrogeomorphology and sources of mortality and morbidity during at early life history stages, that may include stream microbial and insect species.

The overall project objective was to evaluate how inter-trophic level interactions affect lake sturgeon trait variation during early life stages and included the following specific objectives: 1) to quantify the effects of different aquatic insect taxa characterized by different feeding strategies (FS) on lake sturgeon trait variation including egg size, time to hatch, survival to hatch, and body size of free-embryo at hatch (i.e., total length and yolk-sac area), and 2) to quantify the effects of insect taxa of different FS groups on the relative abundance and diversity of microbes on the egg surface, and compare differences in egg surface microbial communities to ambient river water. We hypothesize that egg size, time to hatch, survival to hatch, and body size as well as the composition and abundance of microbial communities will differ as non-lethal and lethal responses to predator exposure during lake sturgeon egg incubation. We further hypothesize that abundance and diversity of egg surface bacterial and lower eukaryotic communities will be lower when eggs are exposed to insects characterized by scraping or grazing feeding strategies.

## Materials and methods

### Study site

The study was conducted in June 2015 at the Black River Streamside Rearing Facility (BR-SRF) located on the upper Black River in Cheboygan County, Michigan, USA (45.3917° N, 84.3328° W). Details are provided [47–49] of the upper Black River study site including spawning habitats and gamete collection methods. Adult lake sturgeon occupy the river annually during multi-modal spawning events between late April and early June [50]. The BR-SRF receives ambient river water (~680 L/min) from Kleber Reservoir [51]. River water was filtered through 100 micron and 50 micron filters prior to experimental use to fertilize and incubate eggs. River water temperature ranged from 15.8°C to 19.5°C (mean, 17.5°C) during experiments.

All methodology used in this study were consistent with Michigan State University standards for accurate and precise reporting and statistical analyses of data.

### Fertilization and incubation

Gametes were collected from a single spawning male and female lake sturgeon in the upper Black River using methods described [49] without use of iodine or other disinfectants or deadhesion compounds. Eggs from a single full-sibling cross reduced variation in egg quality and additive genetic effects [52–54]. Fertilized lake sturgeon eggs were placed into circular 7.62 cm PVC couplings covered with 1 mm^2^ mesh screening, using an average of 34 eggs per coupling. Couplings were briefly placed in a large shallow container used for simultaneous fertilizations using a 1 ml: 200 ml dilution of milt and ambient river water, respectively for several minutes. Eggs and milt were gently mixed inside the container to allow for even fertilization within the couplings. When eggs adhered to the coupling mesh (~1–2 min), the couplings were placed into flow-through (18.9 L/min stream water) Heath Trays for incubation. Successful fertilization was confirmed for all egg groups after 24 hrs using visual staging of developing embryos [55].

### Insect collection

Insects of different FS groups were collected in rock/cobble habitats where lake sturgeon spawn annually [41, 54], and were identified to the genus level. Individuals from aquatic insect families Isonychiidae (genus *Isonychia)*, Perlidae (genus *Claassenia)*, Heptageniidae (primary genus *Stenonema*), and Helicopsychidae (genus *Helicopsyche*) were chosen based on the taxa’s abundance in lake sturgeon benthic spawning habitats [42], and to encompass a range of insect FS groups. We note that other insect taxa including *Nigronia* and *Belostoma* are known predators of early life stages of vertebrates including fishes. However, these and other large predatory insects are not typically found in stream substrates with deposited eggs or free embryo lake sturgeon. Neither are these taxa present is sufficient abundance to negatively impact fish eggs and post-hatch early life stages. Aquatic insects were transported to the BR-SRF and housed in 25.0 L flow-through tanks with a flow rate of 400 mL/min. Insects were held in captivity 24–48 hrs prior to introduction into couplings with fertilized eggs.

### Experimental treatments

Insect experiments were conducted using five treatments: 1) Heptageniidae (general scraper), 2) Perlidae (obligate predator), 3) Isonychiidae (collector-filter/facultative predator), 4) Helicopsychidae (obligate scraper), and 5) a control (no insects) with four replicates of each treatment (20 experimental units). Insects were introduced to the couplings 24 hrs post-fertilization. Multiple couplings were placed in each of six Heath trays within two vertical racks. Coupling placement within and across racks were randomized with respect to insect treatment. Filtered water flow was equally distributed through the vertical Heath incubators, and was maintained at 18.9 L/min until hatch. Because water entering the experimental incubation trays and couplings was filtered at a pore size of 50 microns (see above), the water still contained suspended mater and fine particulates including fine particulate organic matter (FPOM) that is an important insect food source [56]. Insect densities for the Heptageniidae, Perlidae, Isonychiidae, and Helicopsychidae treatments were 5, 1, 5, and 5 individuals per coupling, respectively. Insect densities were estimated using density surveys conducted in gravel substrates at spawning sites in previous years (Scribner unpublished data) [42]. Individuals of approximately equal size, and therefore of similar instar stage, were used across replicates of each insect FS treatment to reduce inter-replicate variability. Insect mortality was monitored daily following insect introduction into the couplings and dead individuals were removed and replaced. No emerging or pupating individuals were observed during the experiment.

### Analyses of egg and free embryo traits

#### Egg and free embryo data collection

Egg samples were collected from each experimental unit 1 day post-fertilization prior to insect introduction (time 1—T1) and 4 days post-fertilization after 3 days of exposure to insects (time 2—T2). Eggs collected from T1 and T2 were photographed and measured using program ImageJ v 1.49 (NIH Image; https://imagej.net/citing) to quantify egg diameter (referred to as egg size). Proportional survival was monitored daily to quantify mean proportional survival at hatch associated with each FS. All couplings were checked once daily to determine the time to 100% hatch (days when all live eggs in the coupling hatched). Hatched free embryos were anesthetized using approved Michigan State University Animal Use and Care protocols (03/14-037-99) with MS-222 and photographed to quantify body size which included total length (TL mm) and yolk-sac area (YSA mm^2^) using ImageJ v 1.49 software.

#### Statistical analysis of trait variation

Each egg incubation coupling was the experimental unit for all response variables used in the analysis. Response variables included mean (±SE) egg size, incubation days to hatch, proportional survival at hatch, body size including total length (TL) and yolk sac area (YSA) at hatch. Analyses were performed using R (4.0.2). Normality was determined using Shapiro-Wilks tests. A general linear model using ANOVA was used to quantify the effects of insects on egg size at times one and two and day to 100% hatch. Mean proportional survival data were analyzed using a generalized linear model fit using a beta distribution. We used Tukey-Kramer multiple pair-wise comparison tests for all response variables and *p* values <0.05 were considered statistically significant.

Body size (TL and YSA) data were correlated, requiring a multivariate approach. However, Mardia’s MVN test for skewness (Statistic = 80.31, p < 0.01) and kurtosis (Statistic = 5.72, p <0.01) indicated that body size data lacked multivariate normality. Additionally, TL and YSA failed to achieve univariate normality and were structured such that data transformations were unsuccessful. To evaluate Mann-Whitney-type effects in nonparametric factorial designs, we used a nonparametric (ranked) MANOVA [57]. Across treatment differences were evaluated using Kruskal-Wallis one-way analysis of variance. Post-hoc evaluations were conducted using Dunn’s Test of multiple comparisons using rank sums [58].

### Molecular characterization of egg surface microbial communities

#### Microbe sampling

Fertilization was confirmed for all eggs prior to introduction of invertebrates into couplings containing eggs. Unfertilized eggs were removed so as not to be confused with mortalities that occurred after invertebrate introduction. Egg status (live vs dead) was determined visually [54] prior to each sampling event (T1 and T2). We subsampled 8 eggs from each replicate from each insect treatment from the screened PVC containers at T1 and T2 to quantify and compare the diversity and relative abundance of microbes on the egg surfaces. Only live eggs were selected based on visual evaluation of development [54]. Developing eggs at each time period (T1 and T2) were preserved in 95% ethanol to preserve bacterial and lower eukaryotic egg surface communities. Microbial genomic DNA was extracted from live eggs from each time period using a modified DNeasy Blood & Tissue QIAGEN Kit protocol (QIAGEN Group, 2006). Modified steps include the initial incubation of samples in an enzymatic lysis buffer at 37⁰C for 30-min followed by bead-beating [59] for 10 min. After bead-beating, steps were followed according to manufacturer’s protocols. To ensure sufficient DNA was extracted for analysis, eight eggs from each sample were pooled during the extraction process [59]. Water samples of 1 L volume were collected at times T1 and T2 using Nalgene collection cups with 0.25 micron filters to serve as a reference of water-borne bacterial and lower eukaryotic communities available for colonization of egg surfaces at each time.

#### 16S and 18S rRNA amplification and sequencing

Polymerase chain reaction (PCR) amplification and sequencing of the v4 region of the 16S rRNA gene was used to estimate the bacterial community composition as a function of exposure to different insect FS groups during incubation. PCR was conducted in a 25 uL reaction volume, containing 5 uL template DNA (13 to 162 ng/uL), 0.25 uL of AquPrime HiFi Taq DNA polymerase (Invitrogen Corp., Carlsbad, CA), 2.5 uL 10X PCR Buffer II, 2.5 uL dNTP (2.5 mM), 0.75 uL MgCl_2_ (50 mM), 0.5 uL 27F Forward Primer (10μm), 0.5 uL 1389R Reverse Primer (10μm), 0.25 uL BSA, and 12.75 uL sterile water. Reactions were performed using the following conditions: initial denaturation step at 95°C for 2 min, then 30 cycles of denaturation at 95°C for 20 sec, annealing at 55°C for 30 sec, and extension at 72°C for 7 min [59]. Samples were submitted for the sequencing at Michigan State University Research Technology Support Facility, (RTSF; https://rtsf.natsci.msu.edu/genomics/; East Lansing, MI, USA). All of the sequencing procedures, including the construction of Illumina sequencing library, emulsion PCR, and MiSeq (v2) paired-end sequencing of the V4 region (~250bp; primer 515F GTGCCAGCMGCCGCGGTAA, and 806R TAATCTWTGGGVHCATCAGGTGCAGG [60] followed [61] adaptation of standard Illumina (San Diego, CA, USA) protocols. Michigan State’s Genomics RTSF provided standard Illumina quality control. Base calling and initial processing (demultiplexing, barcode removal and RTA conversion to FastQ format) was performed using Illumina RTA (v) and Illumina Bcl2Fastq (v).

PCR amplification and sequencing of the coding region for 18S V9 rRNA (~200bp; [62] was conducted using universal eukaryotic primers 1391F (5’-GTACACACCGCCCGTC-3’; [63] and EukB (5’-TGATCCTTCTGCAGGTTCACCTAC-3’; [64]. Primers were selected because of relatively short amplicon size (~200bp), and large taxonomic breadth represented in genomic data bases available in GenBank (NCBI). Samples were sent to the RTSF for DNA sequencing. Creation of sequencing libraries, PCR amplification, and sequencing was performed according to standard Illumina (San Diego, CA, USA) protocols, with 150bp paired-end reads using an Illumina MiSeq sequencing platform.

Details of the bacteria (16SrRNA) and lower eukaryotic (18SrRNA) sequence data analyses pipeline and computational workflow were as follows. Paired-end sequence merging, quality filtering, “denoising", chimera checking, and pre-cluster steps were implemented in program *mothur* v.1.36.1 [60] to develop reference-based taxonomic clustering using the opticluster option in program *mothur*. Operational Taxonomic Unit (OTU) assignment, generally to the genus or occasionally to family level, was performed using the SILVA v SSU 132 [65, 66] reference databases, with clustering sequences defined with 97% identity. Any sequence singletons were removed prior to downstream analyses. Rarefaction analyses were performed to equalize sampling coverage for each sample based on a selected (standardized) sequence depth. To minimize effects of under-sampling while maintaining as broad a dataset as possible, the final OTU assignments represented the lowest classification level attainable based on the SILVA sequence repository. Data were rarefied based on the lowest sample read level.

### Analyses of bacterial and lower eukaryotic community profiles and ecological associations

#### Alpha diversity

All measures of community diversity including OTU richness, evenness, and Shannon and Simpson diversity indices for each egg surface community DNA sample were calculated from the sequence data within *mothur*. Molecular characterizations of microbial communities in river water samples were excluded from statistical analyses but were used in visual comparisons with egg surface communities. Distributions of taxon richness and diversity indices were tested for deviations from normality and assumptions of equal variance among samples by time and treatment variables. If significant deviations from normality or equal variance assumptions were detected, the data was log-transformed or inverse log-transformed as appropriate to meet assumptions.

Analysis of variance (ANOVA) tests were performed on each index of taxon richness and diversity using the *aov* function in R 3.5.1 [67], first testing whether there was a significant interaction effect between time period and invertebrate treatment. If no significant effect was detected, an ANOVA testing for additive effects of time period and treatment of richness/diversity indices was performed. If no significant additive effects were detected, ANOVAs with time period or treatment as the sole explanatory variable were performed. If any ANOVA models indicated significant effects of the explanatory variables, Tukey’s honestly significant difference (HSD) test was performed on treatment variables in the ANOVA model showing a significant effect to determine which groups of samples were significantly different from each other.

#### Beta diversity

To determine if the sampling time period (time T1 vs time T2) and/or insect FS treatments differed in OTU composition of microbial communities, a Permutational MANOVA (PERMANOVA) was performed. 16S (bacterial) and 18S (lower eukaryotic) datasets were analyzed separately, and river water samples were included in these analyses. PERMANOVAs were performed using the *vegan* library in R 3.5.1 [68], using Bray-Curtis distances to estimate pairwise differences between samples with 9999 permutations. PERMANOVAs first tested for an interactive effect between sampling time period and among insect treatments. If no significant interactive effect was detected, a PERMANOVA testing the additive effects of sampling time period and insect treatments was performed.

Hierarchical clustering of samples by community composition was conducted using the *hclust* function in R 3.5.1 [67]. Hierarchical clusters were calculated using the same Bray-Curtis distance matrix supplied to the PERMANOVA models, and clusters were defined by the unweighted pair group method with arithmetic mean (UPGMA). The resulting UPGMA tree was plotted using the *ggtree* function in program R 3.5.1 [69].

Discriminant analysis of principal components (DAPC) was performed on the 16S and 18S datasets to determine if there was variation in microbial community composition that could be used to distinguish between samples from different sampling time periods or insect treatments. DAPC was performed using the *dapc* function in R 3.5.1 [70]. If there was a significant interaction effect between sampling time period and invertebrate treatments identified by the corresponding PERMANOVA, the interaction was used to define prior groups in the DAPC. If no interactive effect was observed, separate DAPCs were performed with sampling time periods or invertebrate treatments to define prior groups. To identify which OTUs contributed the most variation that discriminated between egg communities associated with different insect FS groups, the variance contributed by each OTU to the discriminant functions was taken from each DAPC analysis. All OTUs contributing at least 5% of the total variance were retained and matched to the corresponding taxonomic identification. The OTU count was also correlated with the discriminant function score for each sample to determine which direction on the DAPC plot was associated with higher abundance of the selected OTUs.

#### Quantitative PCR analysis of bacterial community abundance

Quantitative PCR (qPCR) was performed on DNA extracted from bacteria present on 80 egg samples using the universal 16S rRNA gene bacterial primers 331F (5’-TCCTACGGGAGGCAGCAGT-3’) [71] and 519R (5’-GTATTACCGCGGCTGCT -3’) [72] to determine sample bacterial counts. Each 25 μl reaction contained 12.5 μl of Qiagen RT^2^ SYBR® Green ROX™ qPCR Mastermix (Qiagen,Valencia CA), 0.16 μM of each primer and 3 μl of template DNA. For the qPCR reactions, each sample was diluted so that 20 ng of template DNA was present in a 3 μl volume and its dilution factor was recorded. qPCR reactions were run in triplicate on the Applied Biosystems QuantStudio™ 6 Flex Real-Time PCR System (Applied Biosystems, Foster City CA) using the following standard cycling conditions: 1 cycle of 95°C for 10 minutes, and 43 cycles of 95°C for 30 seconds, 60°C for 30 seconds and 72°C for 30 seconds. Fluorescence data were collected during each 72°C extension step.

Serial dilution of a known amount of *Flavobacterium johnsoniae* (accession ATCC 17061) DNA was used to run a standard curve for bacterial quantification for each run [37]. This bacteria taxa possesses six 16S rRNA gene copies in its genome. A standard curve in triplicate was included with each run, and each sample was also run separately three times. By supplying the 16S rRNA gene copy number present in each DNA dilution level within the *Flavobacterium johnsoniae* (accession ATCC 17061) DNA standard curve, the Applied Biosystems QuantStudio™ Real-Time PCR Software calculates the quantity of the 16S rRNA gene copies present in each reaction. This value was multiplied by the dilution factor for each sample to determine the amount of bacteria present in each sample.

## Results

### Egg and free embryo traits

#### Egg size

Mean egg size did not differ significantly among insect FS treatments prior to insect introduction into experimental couplings (T1, *F*_4, 30_ = 0.28, *p* = 0.886; Table 1). This was expected given all eggs were from the same full sibling cross (single adult female and male). However, at T2 (3 da following insect introduction) mean egg size differed significantly among treatments (*F*_4, 30_ = 12.91, *p* < 0.0001; Table 1). Mean (+SE) egg size was smaller in groups exposed to the Perlidae (predator) treatment (3.42 ±0.04 mm) compared to Heptageniidae (scraper) (3.72 ±0.06 mm, *t*_30_ = 4.78, *p* = 0.0015), Helicopsychidae (scraper) (3.65 ±0.02 mm, *t*_30_ = 5.12, *p* = 0.0006), and the control (no insect) (3.72 ±0.06 mm, *t*_30_ = 6.54, *p* < 0.0001) suggesting that predators had removed portions of the outer egg chorion surface. Mean (+SE) egg size in the Isonychiidae treatment (facultative predator/filterer) (3.54 ± 0.03 mm) was significantly smaller than the control (t_30_ = 3.82, *p* = 0.0187). Mean egg size was not significantly different between the control, Heptageniidae (*t*_30_ = 1.76, *p* = 0.7547), and Helicopsychidae (*t*_30_ = 1.41, *p* = 0.913) treatments. The repository for the phenotype data and the bacterial diversity data (see below) can be accessed at https://github.com/ScribnerLab/AquaticInsectLarvalPhenotype/tree/v1.0.0; (doi.org/10.5281/zenodo.6773587).

**Table 1:** Lake sturgeon mean (±SE) egg size (mm) by aquatic insect taxa characterized by different feeding specializations (FS) treatments at Time 2 (4 da post fertilization).

Treatment	FFG	Egg Diameter
Perlidae	Predator	3.42 ± 0.04 x
Isonychiidae	Collector-Filterer	3.54 ± 0.03 x,y
Heptageniidae	Facultative scraper	3.64 ± 0.01 y,z
Helicopsychidae	Obligate scraper	3.65 ± 0.02 y,z
Control	NA	3.72 ± 0.06 z

Values with identical lower case letters are not significantly different (Tukey-Kramer: *p* < 0.05). “NA” indicates the absence of insects (Control).

#### Proportional survival and days to hatch

Lethal and non-lethal effects were observed that differed among insect treatments. Mean (±SE) days to 100% hatch differed significantly among treatments (*F*_4, 15_ = 3.41, *p* = 0.0358). Mean number of days to 100% hatch for free embryos in the Perlidae treatment (5.25 ±0.25 days) was significantly less than those in the Heptageniidae (scraper) treatment (7.00 ±0.0 days, *t*_15_ = 3.34, *p* = 0.0313; Table 2). Mean proportional survival did not vary significantly among treatment groups (*F*_4, 15_ = 1.14, *p* = 0.3745; Table 2). However, survival to hatch for eggs incubated with Perlidae (predators) were >2x lower (0.27±0.10) than eggs incubated with other insect taxa and the control (range of means 0.44 to 0.58; Table 2). We did observe predation on eggs by Perlid predators. In several Perlid replicates eggs were totally consumed (egg counts decreased in couplings during incubation). Further, significantly lower mean egg size in the Perlid treatment suggest that Perlids were ‘removing’ sections of the outer egg chorion surface.

**Table 2:** Mean days (±SE) to hatch and mean proportional survival (±SE) at hatch for lake sturgeon free embryos across aquatic insect feeding specialization (FS) treatments.

Treatment	FS	Mean days to hatch	Mean Proportional survival
Perlidae	Predator	5.25 ± 0.25 x	0.27 ± 0.10 x
Isonychiidae	Collector-Filterer	6.00 ± 0.58 x,y	0.44 ± 0.17 x
Helicopsychidae	Obligate scraper	6.25 ± 0.48 x,y	0.47 ± 0.10 x
Control	NA	6.75 ± 0.25 x,y	0.53 ± 0.11 x
Heptageniidae	Facultative scraper	7.00 ± 0.00 y	0.58 ± 0.15 x

Mean values with identical lower case letters are not significantly different (Tukey-Kramer: *p* < 0.05). “NA” indicates the absence of insects (Control).

#### Free embryo body size and yolk sac area at hatch

In addition to the lethal effects described above, non-lethal effects were observed in the form of ranked effects of total length at hatch and yolk sac area that varied significantly among treatments (Rank MANOVA, ATS = 44.40, p < 0.01, Table 3). Total length at hatch varied significantly among treatments (KW χ^2^ = 34.131, df = 4, p < 0.01 Table 3). Mean total length of free embryos in the Perlidae (predator) treatment (11.33 ±0.15 mm) were significantly smaller compared to free embryos in the Heptageniidae (scraper) treatment (Z = 5.71; p < 0.01) and the control (Z = 4.41, p < 0.01), likely reflecting differences in metabolic activity and treatment differences in time to hatch. Mean YSA of free embryos (KW χ^2^ = 40.10, df = 4, p < 0.01 in the Perlidae (predator) treatment (6.7 ±0.29 mm^2^) were significantly smaller than Isonychiidae (facultative predator, filterer) (Z = 3.63, *p* < 0.01), Heptageniidae (scraper) (7.67 ±0.09 mm^2^, *Z* = 4.86, p < 0.01), Helicopsychidae (scraper) (7.73 ±0.23 mm^2^, Z = 6.04, p < 0.01), and control (7.69 ±0.12 mm^2^, Z = 5.04, p < 0.01) treatments (Table 3). Egg and hatchling data from the facultative predator (Isonychiidae) were intermediate between results from the Perlid and control treatments.

**Table 3:** Lake sturgeon free embryo mean (±SE) total length (TL mm) and yolk-sac area (YSA mm^2^) at hatch across aquatic insect feeding strategy (FS) groups.

Treatment	FFG	Mean TL	Mean YSA
Perlidae	Predator	11.33 ± 0.15 x	6.70 ± 0.29 x
Isonychiidae	Filterer/Facultative predator	12.02 ± 0.49 y	7.63 ± 0.08 y
Helicopsychidae	Obligate scraper	12.58 ± 0.34 y	7.73 ± 0.23 y
Control	NA	12.71 ± 0.17 y	7.69 ± 0.12 y
Heptageniidae	Facultative scraper	12.90 ± 0.23 y	7.67 ± 0.09 y

Values in the TL and YSA columns with identical lower-case letters are not significantly different (Dunn’s Test: *p-adjusted Bonferroni* < 0.05). “NA” indicates the absence of insects (Control).

### Molecular characterization of egg surface microbial communities

#### Summary of library egg surface microbial taxonomy

Metabarcoding analysis of egg surface bacterial (16S) and lower eukaryotic (18S) samples successfully amplified sequences from 39 and 40 experiment groups of eggs, respectively across all insect treatments, as well as communities from water samples during each time period. Water samples from the river at the point of entry to experimental incubation trays in the research facility were collected during T1 and T2 and sequenced. Raw sequence reads were deposited to the NCBI Sequence Reads Archive (SRA) under BioProject accession number PRJNA855298 (for bacterial 16S data) and PRJNA855303 (for lower eukaryotic 18S data).

Samples sequenced for the bacterial 16S region produced 5.51M reads, were rarified to 5089 sequences per sample containing 2821 unique bacterial OTUs with >1 read. The most abundant 16S sequences from egg surface OTUs (means over all samples per treatment) were from the families Comamonadaceae (2 OTUs 16.1% and 2.3%), Pseudoalteromonadaceae (3.4%), Burkholderiales (2.9%), Pasteurellaceae (2.4%), Rhodobacteraceae (2.5%), Moraxellaceae (2.2%), and Methylophilaceae (2.1%). Water samples from T1 and T2 shared the vast majority of bacterial OTUs found on egg surfaces with the exception of three genera in the families Moraxellacae and Burkholderiacae (*Pseudoalteromas*, *Enhydrobacter*, and *Ralstonia*) that were in the water but not on egg surfaces. The repository for the bacterial 16S taxonomic community matrix can be accessed at https://github.com/ScribnerLab/AquaticInsect16sCommunityMatrix/tree/v1.0.0; doi.org/10.5281/zenodo.6773583.

Samples sequenced for 18S produced 980,850 reads, were rarified to 8348 sequences per sample and contained 1021 unique OTUs with >1 read. Across all 40 lake sturgeon egg samples, an average of 91% of the sequences were from lake sturgeon (range 88.4 to 98.8%). Of the non-fish lower eukaryote OTUs, the most abundant 18S sequences from lake sturgeon egg surface OTUs (means over all samples) included the oomycete genus *Saprolegnia* (11.4%), a filamentous true fungi (subkingdom Dikarya Basidiomycota; 21.6%), and a ciliated protist (Conthreep; 4.1%). The remaining OTUs could not be classified. Water samples from T1 and T2 shared the vast majority of lower eukaryotic OTUs found on egg surfaces with the exceptions of two OTUs in the Eumetazoa and Dikarya. The repository for the lower eukaryotic 18S taxonomic community matrix can be accessed at https://github.com/ScribnerLab/AquaticInsect18sCommunityMatrix. doi.org/10.5281/zenodo.6783566.

#### 16S rRNA (v4) bacterial alpha diversity

**Fig 1:**
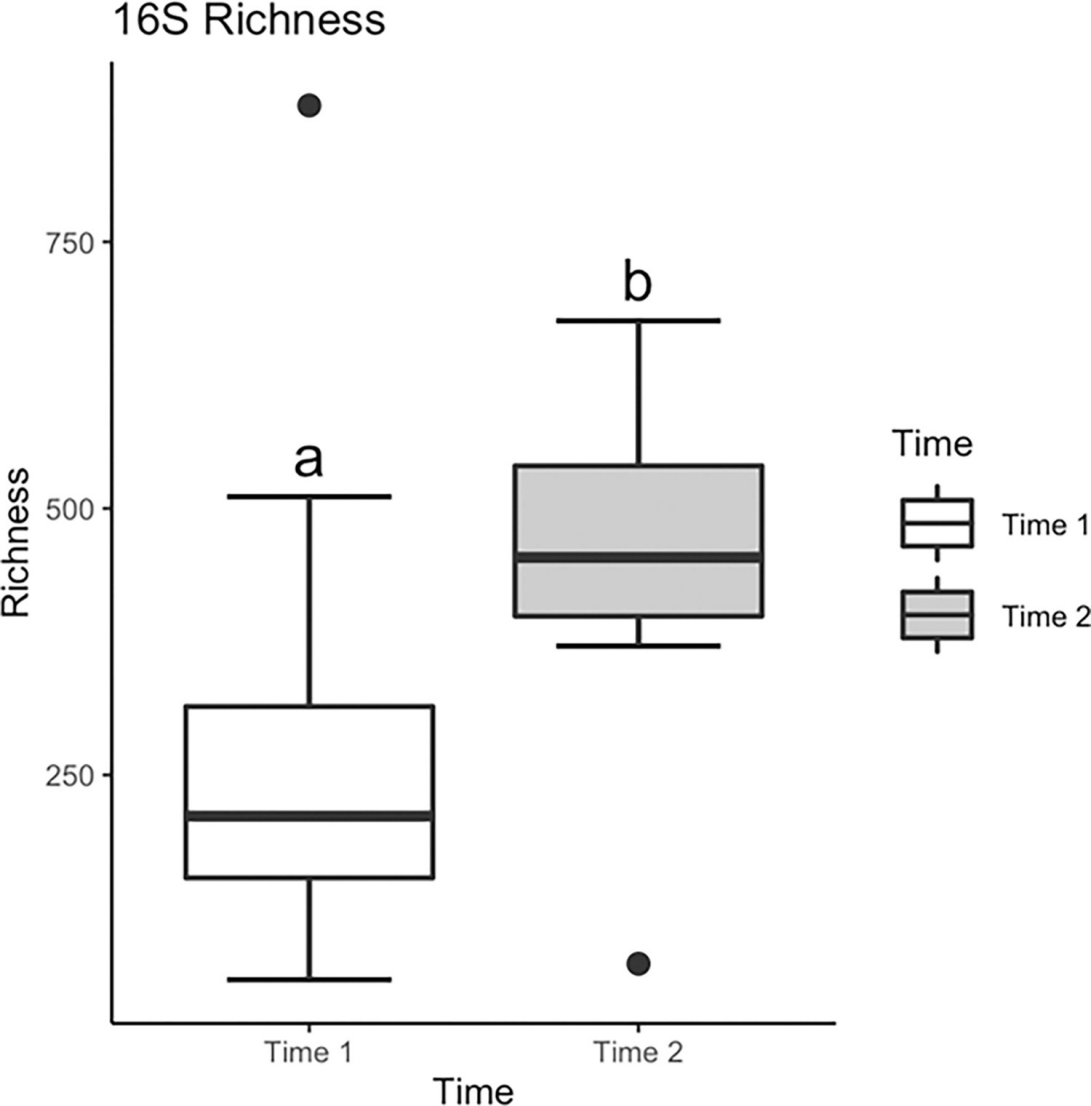
Bacterial (16S) lake sturgeon egg surface community richness (means±SE) for samples across aquatic insect treatments collected during time 1 and 2 (1 da and 4 da post fertilization, respectively).

**Fig 2:**
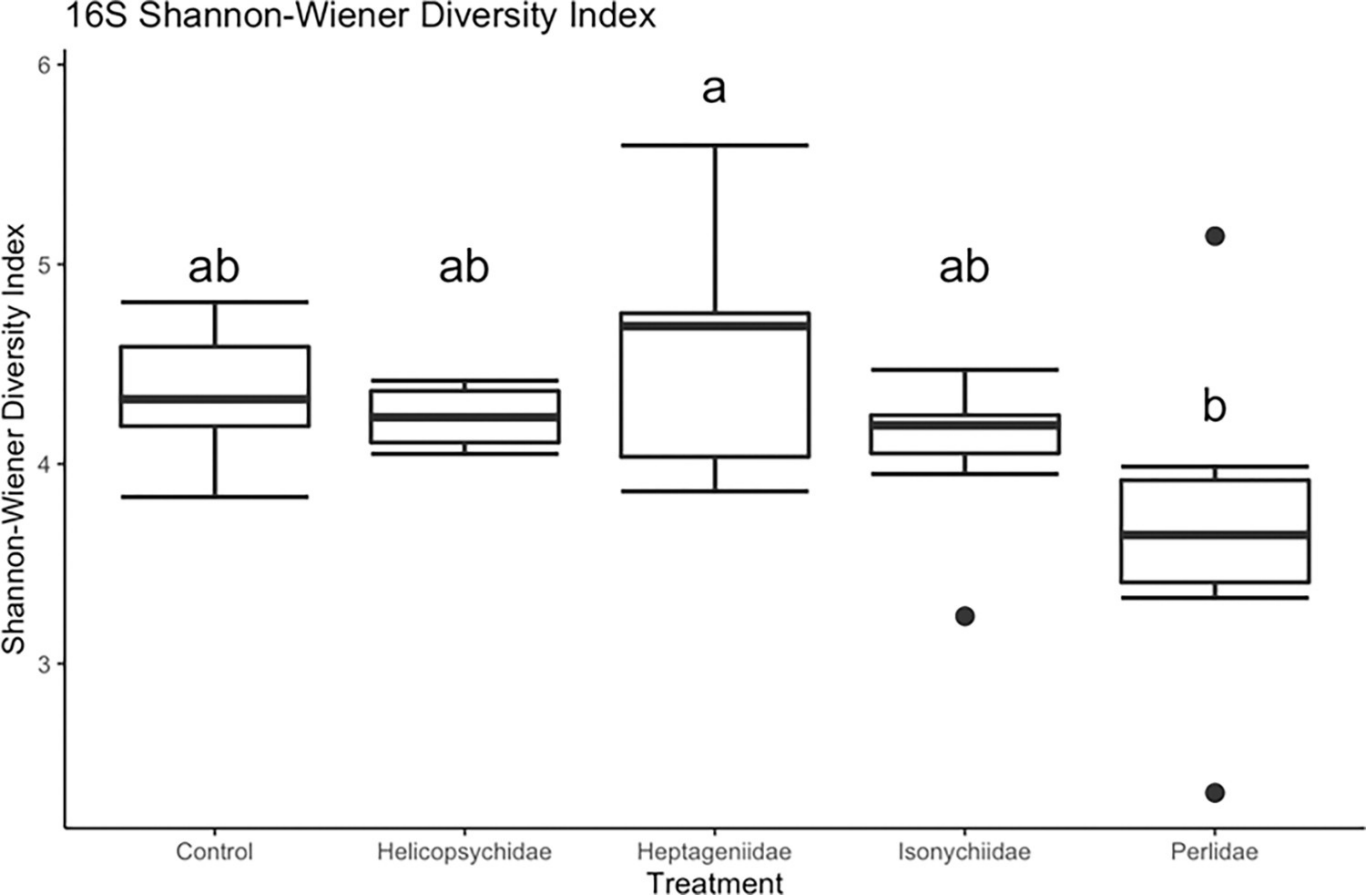
Bacterial (16S) Shannon diversity estimated for lake sturgeon egg surface communities from aquatic insect treatments during time 2 (4 da post fertilization).

Mean bacterial OTU richness (OTU number) did not vary significantly among insect FS treatments, however differences in OTU richness were evident between the sampling times (F_1, 29_ = 13.527, *p* = 0.0009), with richness being nearly twice the level during T2 than T1 (Fig 1). Mean Shannon bacteria diversity varied significantly among insect treatments (F_4, 34_ = 3.19, *p* = 0.025; Fig 2). Mean Shannon diversity of bacteria was significantly lower in eggs incubated with Perlidae (predator) (Tukey multiple comparison of means, *p* = 0.017). Similar to Shannon diversity, mean Simpson bacterial diversity varied significantly among insect treatments (F_4, 33_ = 3.354, *p* = 0.0207). Mean Simpson microbial diversity was significantly lower in groups incubated with Perlidae (predator) (Tukey multiple comparison of means, *p* = 0.009).

**Fig 3:**
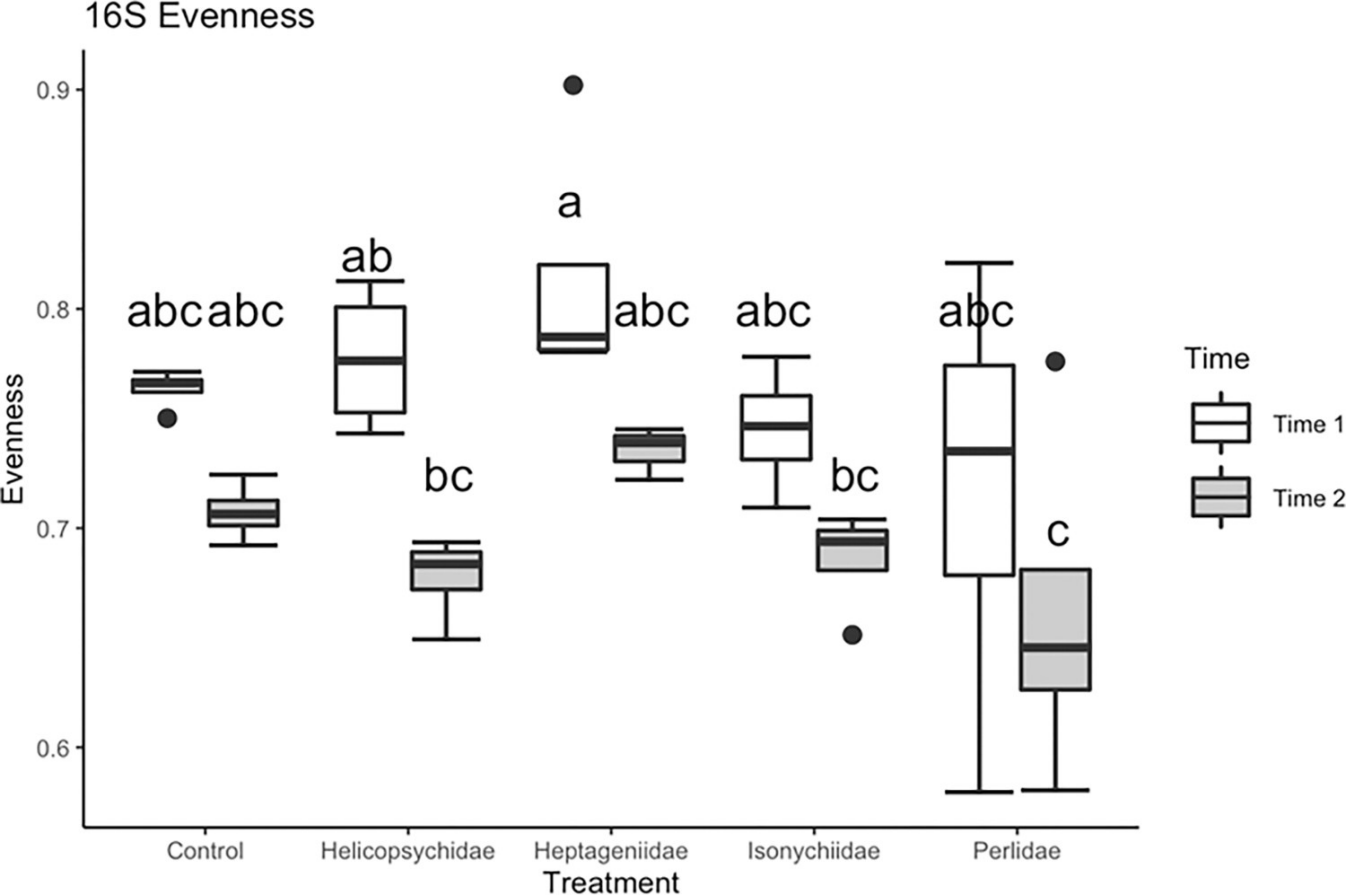
Bacterial (16S) lake sturgeon egg surface community evenness estimated for all aquatic insect treatments during time 1 and time 2 (1 da and 4 da post fertilization, respectively).

Time (F_1, 33_ = 20.87, P<0.0001) and insect treatment (F_4, 33_ = 3.61, P = 0.015) had significant effects on bacterial OTU evenness (degree of equitability in OTU relative abundance, Fig 3). Egg surface bacterial communities prior to the introduction of insects (T1) had greater evenness than eggs at 3 da following insect introduction (T2). Heptageniidae (scraper) generally had a neutral effect on bacterial community evenness (relative to the no insect control) while Perlids (predators) generally had a negative effect on evenness with bacterial communities being dominated by a few taxa compared to the other treatments (Fig 3). Helicopsychid (scraper) and Isonychiid (facultative predator/filterer) treatments were also trending lower but were not significant.

#### qPCR data on bacterial 16S copy number

**Fig 4:**
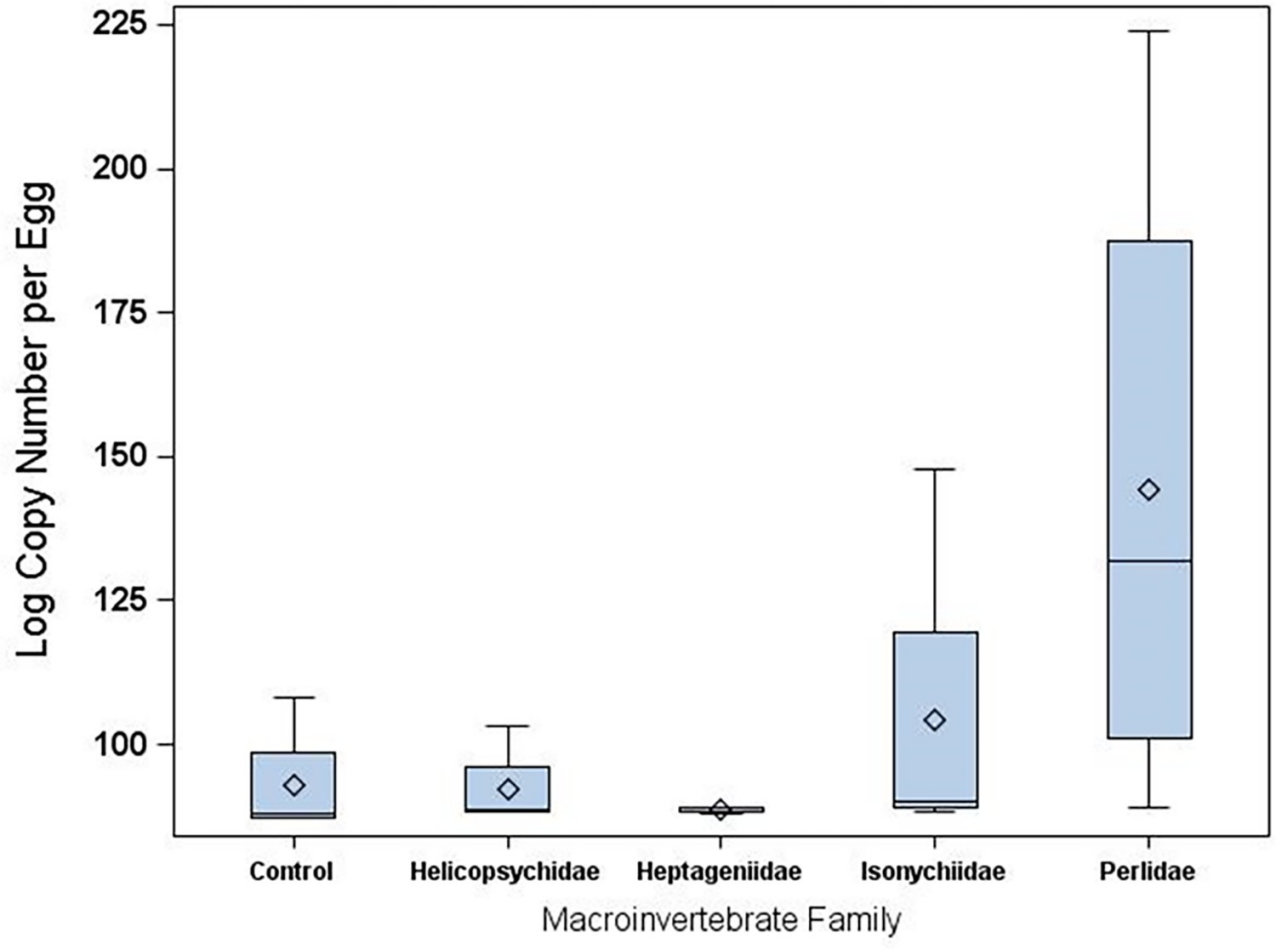
Quantitative PCR analyses of bacterial (16S) abundance for lake sturgeon egg surface communities sampled from each aquatic insect treatment during time 2 (4 da post fertilization).

Levels of 16S copy number varied greatly among invertebrate treatments (F_4, 15_ = 5.24, *p* = 0.0076; Fig 4). Higher 16S copy number (and inferentially bacterial abundance) was observed in the Perlidae treatment than for egg surface bacterial communities exposed to Heptageniidae (scraper), Isonychiidae (facultative predator/filterer), Helicospsychidae (scraper), or controls (no invertebrates (Fig 4).

#### 18S (V9) lower eukaryotic alpha diversity

**Fig 5:**
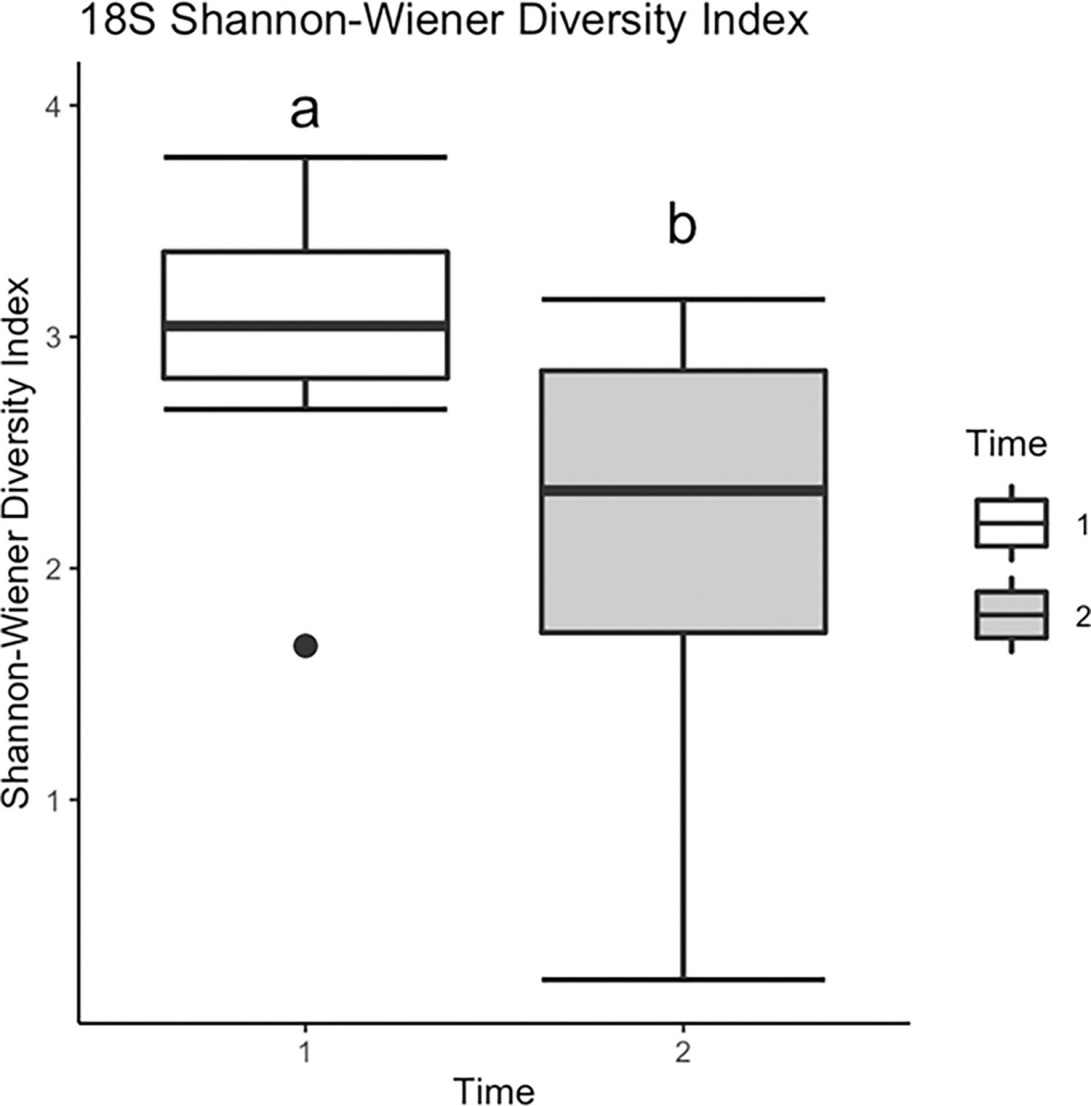
Lower eukaryotic (18S) Shannon diversity estimates (mean±SE) contrasting egg surface communities across aquatic insect treatments sampled during times 1 and 2 (1 da and 4 da post fertilization, respectively).

**Fig 6:**
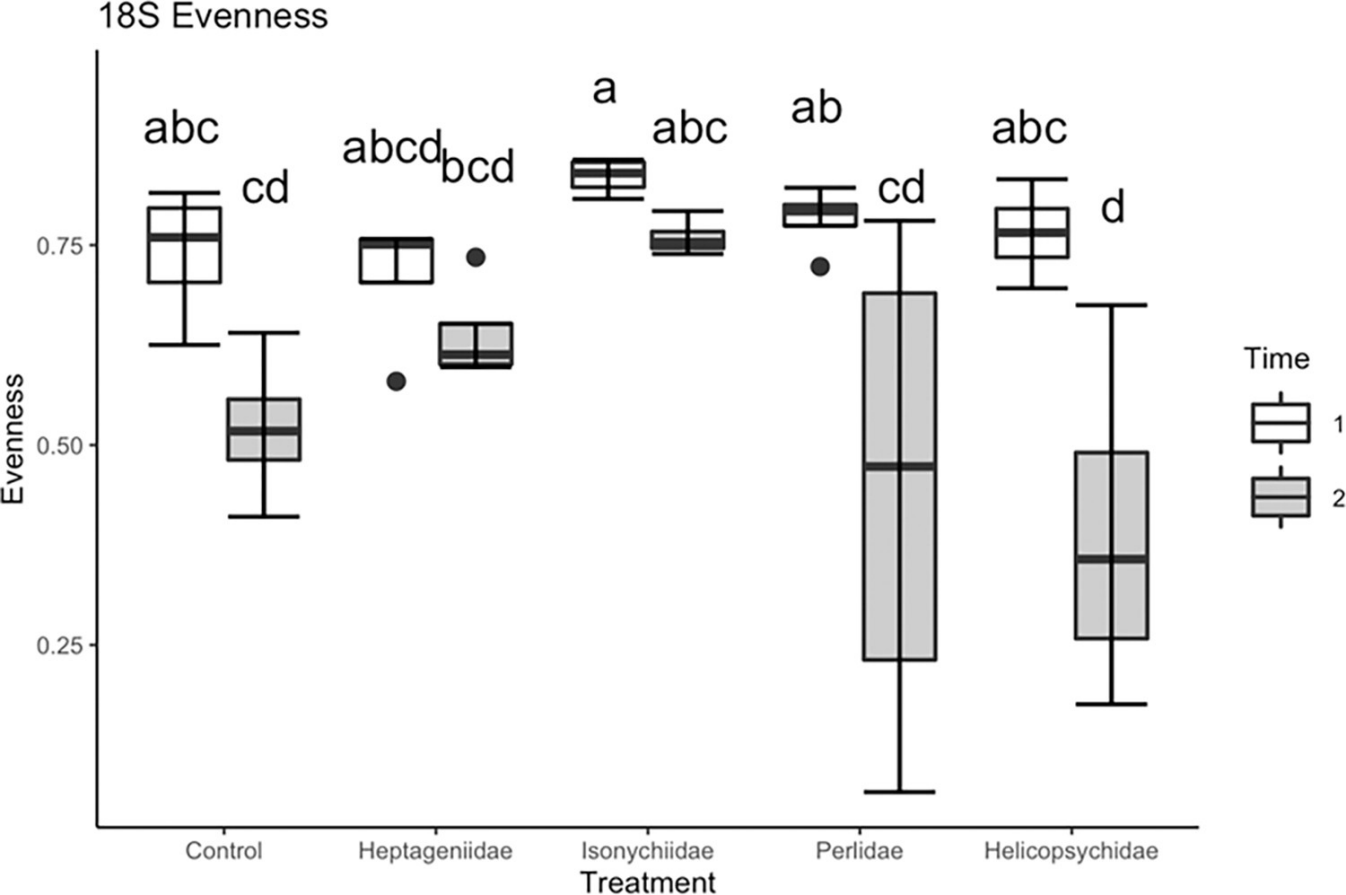
Lower eukaryotic (18S) lake sturgeon egg surface community evenness (means±SE) for estimated across aquatic insect treatments during time 1 and time 2 (1 da and 4 da post fertilization, respectively).

Levels of lower eukaryotic (18S) egg surface community diversity showed the opposite trend relative to bacterial (16S) diversity, where Shannon-Wiener diversity was lower during T2 relative to T1 (F_1,38_ = 19.44, *p<*0.001; Fig 5). However, mean Shannon microbial diversity did not vary significantly among treatment groups (F_4, 15_ = 2.01, p = 0.1441). Mean Simpson microbial diversity also did not vary significantly among treatment groups (F_4, 15_ = 2.45, p = 0.0917). Mean egg 18S community evenness differed significantly between time periods F_1,34_ = 33.06, p<0.0001; Fig 6) and among treatments (F_4,30_ = 4.48, p = 0.0052; Fig 6). Lower eukaryotic (18S) community evenness declined significantly over time for all treatments, and decreases were particularly pronounced on eggs incubated with Perlidae (predators) and Helicopsychidae (scrapers) (Fig 6).

#### 16S bacterial beta diversity

**Fig 7:**
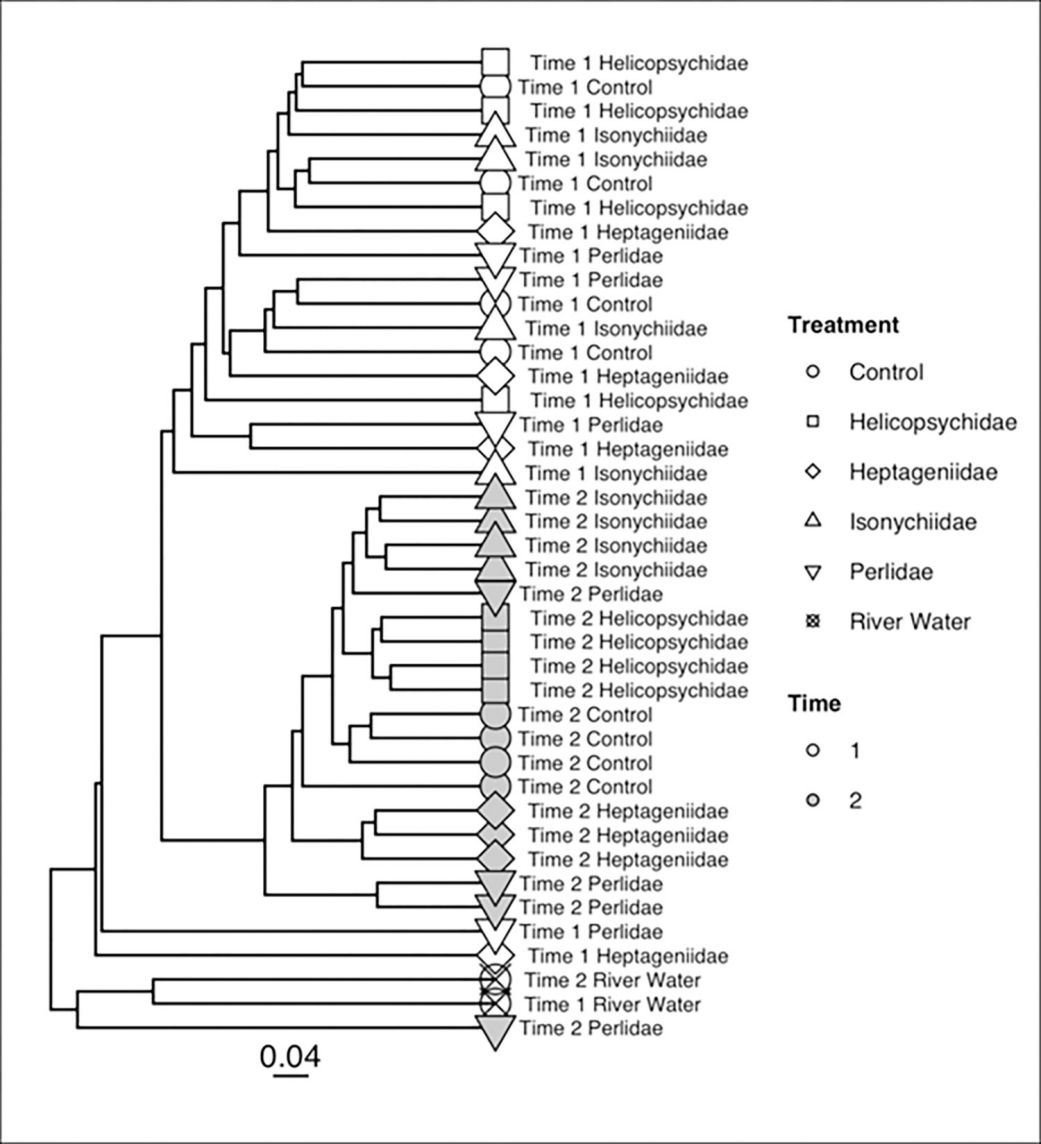
UPGMA diagram based on bacterial (16S) Bray-Curtis distances among lake sturgeon egg surface communities sampled during two time periods for each of 4 aquatic insect treatments, a no insect control, and stream water. Time 1 and Time 2 refer to 1 da and 4 da post fertilization, respectively.

PERMANOVA models detected significant additive effects of both sampling time period (PERMANOVA, pseudo-F = 8.716, p = 0.0001) and insect treatments (PERMANOVA, pseudo-F = 2.474, p = 0.0001) on 16S microbial community composition. No significant interaction effects were detected (pseudo-F = 1.30, p = 0.088). Community taxonomic differences among samples based on Bray-Curtis distance are visualized in the UPGMA tree (Fig 7). Most samples from the same sampling time period clustered together. Clustering by insect treatment was apparent in T2, though the Bray-Curtis distances between the 16S microbial communities in T2 were overall lower than in T1. River water samples clustered together with one of the Perlidae treatment samples from T2 to form an outgroup relative to the rest of the egg 16S microbial communities, indicating non-random colonization from water to egg surfaces.

**Fig 8:**
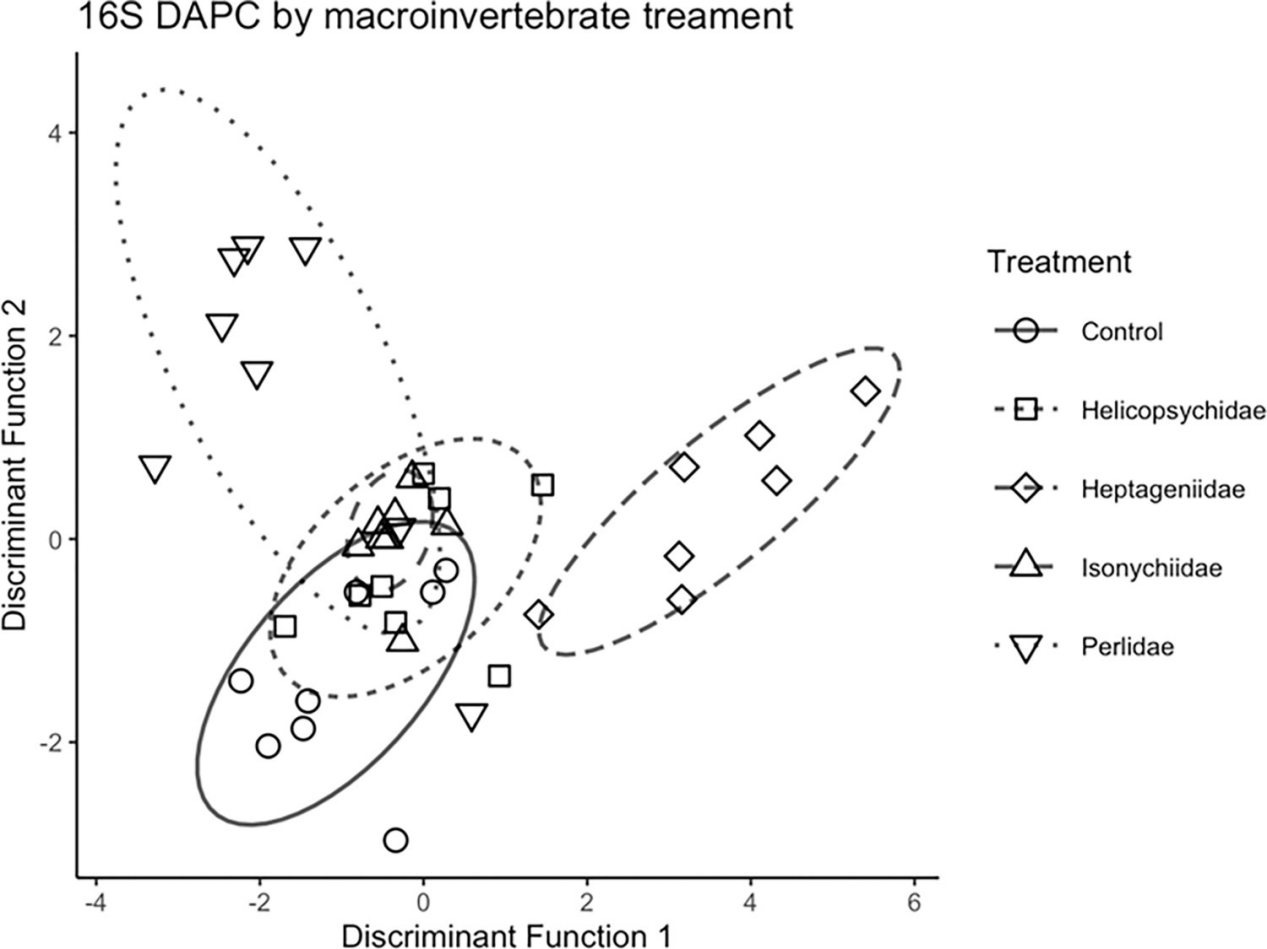
Multivariate ordinations of lake sturgeon egg surface bacterial (16S) communities based on discriminant analysis of principle components using Bray-Curtis distances for aquatic insect treatments sampled during time 2 (4 da post fertilization).

Because the 16S microbial community PERMANOVA model indicated additive effects of insect treatments and sampling time, these variables were analyzed using DAPC separately. With invertebrate treatments as prior groups, two discriminant functions were produced (T2 communities; Fig 8). The first discriminant function clearly discriminated between the river water samples and the egg surface samples. There were five OTUs that contributed >5% of the variance used in the first discriminant function (S1 Table), four of which were more common in the river water samples (*Pseudoalteromonas*, *Ralstonia*, *Enhydobacter*, and *Streptococcus*) and one of which was mostly found on egg surfaces (*Acinetobacter*). The second discriminant function was able to discriminate between some of the Perlidae (predator) treatment samples and the Heptageniidae (scraper) treatment samples. There were four OTUs that contributed >5% of the variance used in the second discriminant function (S2 Table), all of which were more common in the Perlidae (predator) treatment samples. There were five OTUs that contributed >5% of the variation used in the discriminant function to discern between samples from T1 and T2 (S3 Table). One OTU (Pasteurellaceae) was more common in time T2 than T1 while four OTUs were more prevalent in T2 (Burkolderiales, Rhodobacteraceae, *Ralstonia*, and an OTU identified as *Fluviicola* that was responsible for 27% of the variation used in the discriminant function.

#### 18S lower eukaryotic beta diversity

**Fig 9:**
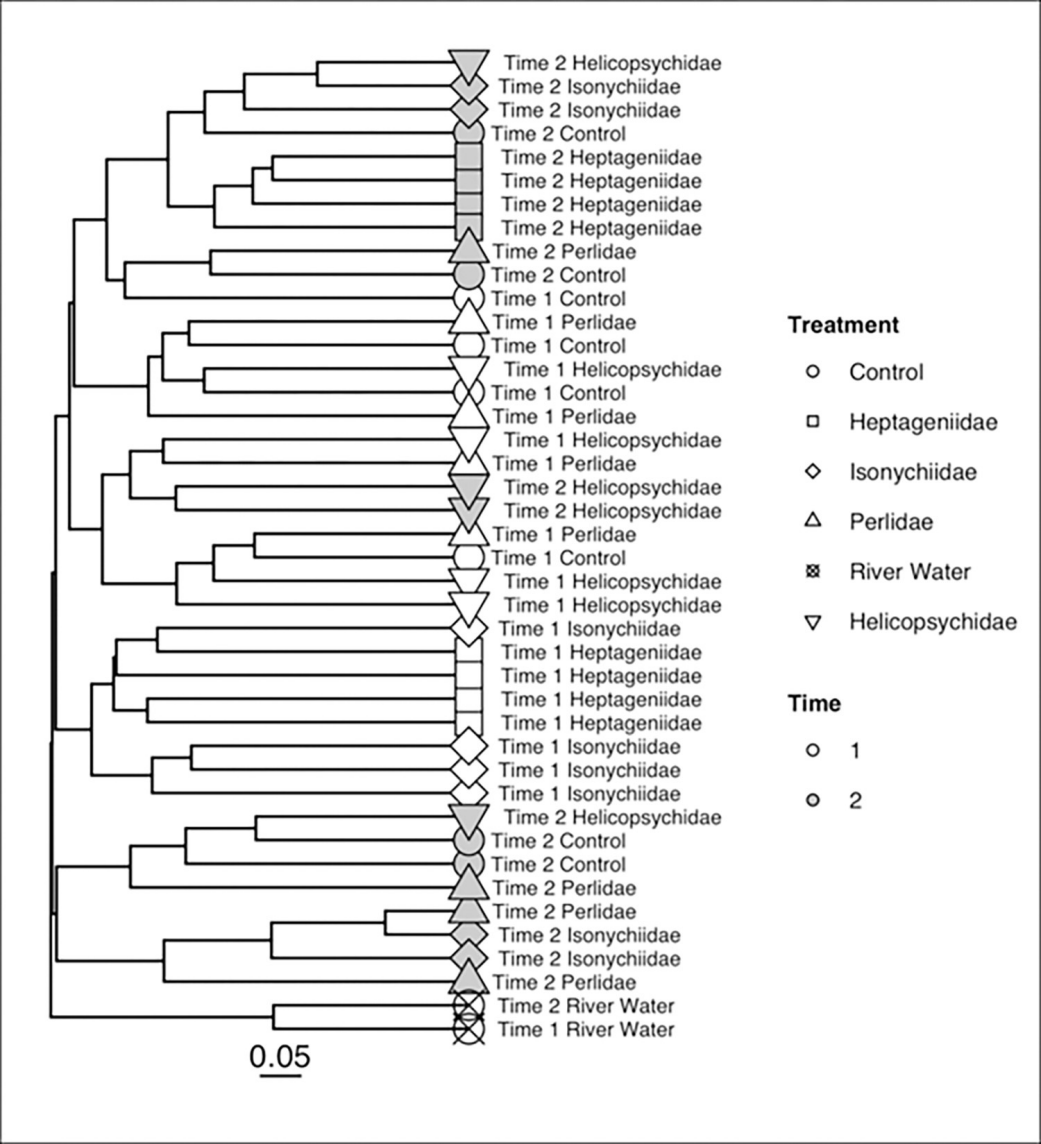
UPGMA diagram based on lower eukaryotic (18S) Bray-Curtis distances among lake sturgeon egg surface communities sampled at two time points for each of 4 aquatic insect treatments, a control (no insect) and stream water. Time 1 refers to collections 1 da post fertilization and Time 2 refers to 4 da post fertilization.

The Bray-Curtis distances of 18S OTU community taxonomic composition were also visualized with a UPGMA tree (Fig 9). Lower eukaryotic communities from water sampled collected during T1 and T2 were distinct from the egg surface communities, as described for bacterial communities, again indicating non-random community colonization. Lower eukaryotic communities clustered by sampling time (Fig 9). However, there were two distinct clusters of samples from T2 that generally grouped by insect FS treatment. PERMANOVA documented a significant interaction between treatment and time (pseudo F = 1.24, R^2^ = 0.119, p = 0.035), so the interaction of these variables was analyzed in the DAPC. The first two discriminant functions described 95% of the variation that differentiates the communities of different treatments from T1 to T2. Nearly all of that variation was due to how different the river water communities were from communities on the egg samples (discriminant function 1). The second discriminant function was mainly associated with the difference between the communities from the river water samples from T1 to T2 (Fig 9).

**Fig 10:**
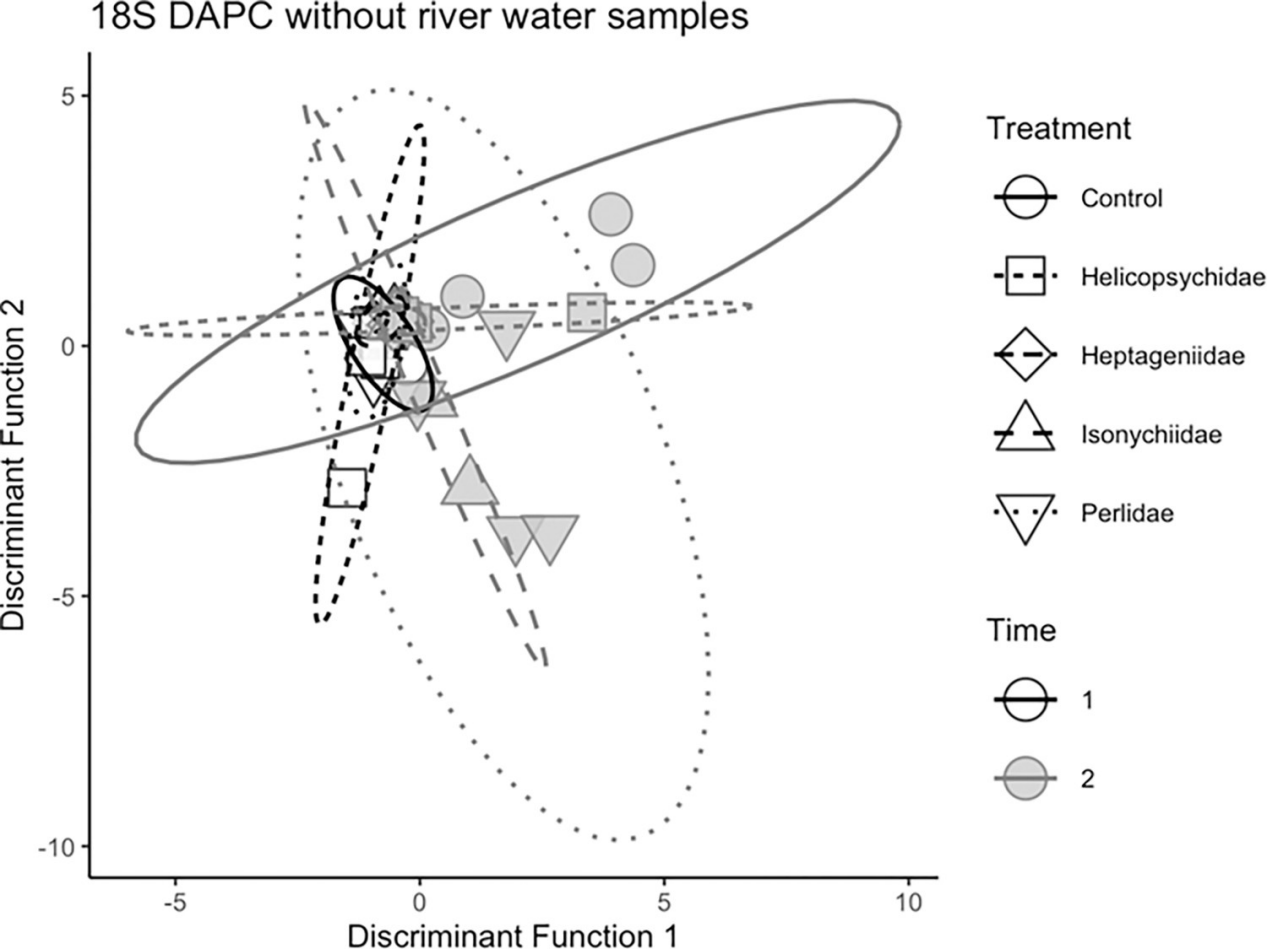
Multivariate ordinations of lake sturgeon sturgeon egg surface lower eukaryotic (18S) communities based on discriminant analysis of principle components using Bray-Curtis distances for aquatic insect treatments sampled during time 1 and time 2 (1 da and 4 da post fertilization, respectively).

Because most of the variation described in the 18S DAPC was associated with the river water samples, another DAPC excluding the river water samples was performed to determine whether there was variation that could be used to discriminate between the egg communities. Two discriminant functions were retained from this DAPC (Fig 10). The first discriminant function was associated with differences in the communities from some of the T2 Control, Perlidae, and Isonychiidae samples from the rest of the egg samples. There were four OTUs that contributed >5% of the variation used in the first discriminant function (S4 Table), all of which were more common in the T2 Control, Perlidae (predator), and Isonychiidae (facultative predator) samples. The second discriminant function discriminated between the T2 Control samples, and the T2 Perlidae and Isonychiidae samples. There were three OTUs that contributed >5% of the variation in the second discriminant function (S4 Table), all three of which also contributed >5% of the variation to the first discriminant function as well. One identified as *Saprolegnia* was more common in the Control samples and two identified as Holozoa and Peronosporomycetes more common in the Isonychiidae and Perlidae treatment samples.

## Discussion

Interactions among fishes, aquatic insects, and diverse members of bacterial and lower eukaryotic communities are a critical focus area for studies of physical and biotic factors in stream environments that create barriers to recruitment for fish populations. In this study, lake sturgeon eggs were experimentally exposed to members of one of four abundant stream-resident insect taxa. We hypothesized that selected aquatic insects, each characterized by different feeding strategies (FS), would have lethal and non-lethal effects on lake sturgeon eggs and newly hatched free embryos, including effects mediated through modification of egg surface microbial community abundance and taxonomic composition.

### Lethal and non-lethal interactions between insects and lake sturgeon during early life stages

Predation is one of the leading causes of fish egg and larval mortality. Consequently, predation can negatively impact population levels of recruitment of even the most fecund fish species [73]. Vulnerability to predation increases due to the aggregated distribution and limited mobility of fish eggs and larvae [40], which is particularly notable in broadcast spawning species like lake sturgeon. Additionally, small egg size makes eggs accessible to a wide range of predators, including macroinvertebrates [74, 75]. We documented lethal effects on lake sturgeon eggs by Perlid predators based on two-fold lower survival of eggs exposed to Perlid predators during incubation (Table 3) relative to other insect treatments and the control. In several Perlid replicates, eggs were totally consumed (egg counts decreased in couplings during incubation). Further, mean egg size in the Perlid treatment was significantly lower (Table 1) relative to other insect treatments and the control, suggesting Perlids were removing or otherwise compromising the egg chorion surface.

Following hatch, lake sturgeon early-life stages are characterized by high rates of mortality [76]. Newly-hatched free embryos lack physical and sensory structures [13], and as a consequence, at hatch they burrow in stream substrate [77], and remain in substrate interstitial spaces until endogenous yolk resources are absorbed [75]. Larvae emerge from stream substrate and disperse to depositional downstream regions of the stream to exogenously feed [78]. Previously, we have documented plasticity in emergence associated with stream physical environmental conditions (temperature and discharge, [79]) and with predator cues [45]. Here we quantify a high degree of behavioral and physiological plasticity in response to non-lethal effects of Perlid predators and to a lesser extent by facultative predatory Isonychiidae. The family Isonychiidae consisted of one genus in Michigan, *Isonychia*, which are defined as both collector-filterers and facultative predators [16]. When isolated with lake sturgeon eggs and filtered water, *Isonychia* nymphs may switch their food source from the filtered, resource-poor hatchery water to lake sturgeon eggs. The two feeding behaviors that are represented in this mayfly genus could explain the consistent intermediate effects on most variables measured in this experiment.

Lake sturgeon responded to presence of Perlids by hatching significantly earlier (by one or more days) than eggs exposed to other insect taxa and the control (Table 1). Accelerated time to hatch was associated with a significant reduction in newly hatched free embryo body size (TL), and accelerated use of yolk sac reserves reflected in smaller yolk sac area at hatch (Table 3). As seen in this study and previously, early life stress [80] during incubation including non-lethal predator effects [23, 24, 79, 81], can impact individual behavior and physiology during subsequent life stages that has implications for future survival [82].

Early hatching as a method for predator evasion has been observed across a wide range of taxa [83], including fish [27]. Data reported here support our hypothesis that egg-bound lake sturgeon embryos may detect predators during incubation and respond by hatching earlier. However, the mechanism associated with predator detection and response by lake sturgeon at the egg stage was not tested. Numerous causes could be involved (review in [84]), including odorant cues released by prey [85] such as conspecific signaling [86, 87], chemosensory perception of cues released by predators [88] or disturbance cues [89]. Studies of plasticity in hatch time in response to predators suggest that an olfactory mechanism is possible [27, 29]. The development of olfactory epithelium in the family Acipenseridae is similar to that in the Anuran genus *Xenopus* [30]. Several members of the order Anura have exhibited an early hatch response to predatory chemical cues [90–94] documented early hatching and altered morphologies of prey after chemical contact with the predator, inferring that chemical cue detection at the egg stage is advantageous. However, predator detection mechanisms and subsequent plastic responses vary among taxa. For example, authors [95] documented egg-phase fish responding to vibratory cues which resulted in early hatching. Predators could compromise the integrity of egg membrane surfaces and allow embryos to escape egg capsules. Evidence of Perlid disruption of the egg chorion surface, seen as reductions in egg size indicates that tactile stimuli may be involved in early free embryo hatch.

Changes in morphology of fish and other taxa resulting from exposure to predators during early life stages can have consequences during subsequent life stages [27, 83, 93, 94, 96]. Responses vary across taxa, and are dependent on the predator community [97]. For example, early hatching in response to predator detection may be initially advantageous by avoiding consumption during the vulnerable, immotile egg stage.

Lake sturgeon adults provide no post-ovulatory parental care. Accordingly, conditions encountered by offspring during incubation, and maternal effects are important to growth and survival [98]. The period where larvae shift from endogenous reserves to exogenous feeding is particularly critical for survival [13, 99], due to the risk of predation and starvation at these early life stages [100]. Both sources of mortality have been tied to body size. Larger young fish are better able to gather food, survive periods of low resource abundance, and are better able to escape predators [13, 101]. If ‘bigger is better’, then eggs hatching at a smaller embryonic body size and with lower levels of yolk resources, as documented for free embryos associated with the Perlid treatment (Table 3), could experience higher levels of mortality following exposure to Perlids during incubation. Hatching at a small size may increase susceptibility to predators that are gape-limited or those that prefer smaller prey [102]. Findings from previous studies that predation rates decrease with increasing larval body size have been widely reported [103–106]. This observation is consistent with mesocosm experiments investigating predation rates of rock bass (*Ambloplites rupestris*) on larval lake sturgeon from the Upper Black River [107]. Findings of size selectivity of individuals of different life stages with and without alternative prey present have also been documented [e.g., 108, 109].

Alternatively, early hatching at a smaller body size could allow free embryos access to smaller interstitial spaces in the stream beds, potentially decreasing the chance of predation. Furthermore, hatching at a smaller body size can also allow lake sturgeon free embryos to temporarily avoid the preferred prey size class of a predator. For example, [102] demonstrated that white sturgeon (*Acipenser transmontanus*) free embryos preferred hiding in substrate comprised of small gravel as this resulted in decreased predation by sculpins (*Cottus* spp.). Hatching at a smaller size may provide additional refuge for free embryos. Authors [110] found that certain predators choose larger fish larvae. Our experimental design did not allow us to determine the mechanism for predator detection in developing lake sturgeon eggs, and consequences during subsequent life stages. Results from this experiment warrants further investigation of the fitness consequences of free embryos at a smaller body size in the presence of predatory insects and fish. Future research could profitably investigate trade-offs associated with early hatching at a smaller size while considering the composition, function, and prey preference of the predator community.

### Associations between microbes and lake sturgeon eggs mediated by aquatic insects

Microbes are ubiquitous in aquatic environments and are found as free suspended and benthically drifting forms and associated with biofilms, and vary in composition and abundance spatially and temporally [111]. Stream biofilms are biologically complex and are associated with aquatic surfaces, predominately on bottom substrates [34]. Aquatic microbial populations form an abundant resource that are widely used by many aquatic insect taxa [112] and promote insect fitness [113]. At this trophic level, emerging information [37] suggests that microbial populations can have either beneficial or detrimental effects during early lake sturgeon life stages.

In this study, sampling time and insect FS treatments had marked effects on bacterial (characterized by 16S sequences) and lower eukaryotic (characterized by18S sequences) microbial communities. Previous studies have shown that there are successional patterns seen in the egg surface bacterial community [59], which were also observed in this study. For both bacterial and lower eukaryotic communities, a general pattern was observed where overall evenness declined between fertilization (T1) and three days following insect introduction (T2), as a few taxa became more abundant (Figs 3 and 6) to dominate the microbial community, causing measures of diversity to remain steady or decline, even as the total number of unique taxa present on the egg surface stayed constant or increased. The diversity of bacterial and lower eukaryotic communities responded similarly to the insect FS treatments. Eggs placed in the Heptageniidae (general scrapers) treatment generally exhibited an increased level of evenness and diversity compared to eggs in the control treatment while eggs in the predator treatment exhibited decreased levels of evenness and diversity. Microbial community diversity is often associated with reduced risk of invasibility [114]. Diverse microbial communities likely play important roles in protecting hosts from potential pathogens, as seen in well-studied pathogen-host systems in amphibians [115], and fish [32]. This study also found that less diverse microbial communities had higher abundances of taxa known to contain fish pathogens, with *Saprolegnia* becoming more common in the predator FS treatments (S4 Table).

Results indicated that grazing/scraper insects fed on bacterial or other lower level communities growing on eggs. Evidence for feeding by members of any feeding group are based on measures of diversity based on 18S and 16S sequences, bacterial copy number (based on qPCR), and compositional heterogeneity (UPGMA dendrogram showing community differences based on Bray-Curtis distance). For example, Fig 6, depicts a decrease in lower eukaryotic taxon evenness between T1 and T2. That decline is particularly prominent on eggs exposed to the Perlidae and Helicopsychidae treatments. These results likely indicate taxonomic alteration potentially involving selective grazing. In the UPGMA dendrogram we see that during T2, replicates from all the insect treatments cluster together, also suggesting taxon-specific selective grazing.

The underlying community dynamics were revealed by molecular characterization of community composition. There appears to be affiliations of specific microbial taxa to egg surfaces, as the taxonomic composition of microbial communities on egg surfaces differ substantially from the microbial community composition of river water (Figs 7 and 9 for bacteria and lower eukaryotes, respectively). Successional patterns were one of the strongest influences on the bacterial community composition of egg surfaces, with several of the most common OTUs (Burkholderiales, Rhodobacteraceae, Fluviicola) only becoming highly abundant during the later sampling period (T2). These taxa were drivers in the reduction of evenness seen from T1 to T2. The lower eukaryotic community also showed evidence of a successional pattern, with *Saprolegnia* becoming the dominant OTU during T2, though *Saprolegnia* relative (sequence) abundance was also strongly influenced by the invertebrate treatment.

Insect FS treatments played varied roles in mediating microbial community taxonomic composition during the first 3 days of the incubation period. Heptageniidae and Perlidae had the greatest effects on bacterial communities. Heptageniidae treatments generally had the lowest abundance of Comamondaceae. Some Comamondaceae are components of periphyton, a main food source for Heptageniidae larvae [116, 117], indicating that the scraper FS taxa may be feeding on the egg surface microbial community and preventing Comamondaceae from dominating the community. Meanwhile, the predator Perlidae, and to a lesser extent, Isonychiidae, had less consistent, but generally more disruptive effects on bacterial and lower eukaryotic microbial communities. The bacterial communities of egg surfaces from the Perlidae treatment were more likely to become dominated by taxa also present in river water samples. Results suggest that predators may compromise eggs surface integrity (e.g., egg size reduction; Table 1), thereby allowing recurrent colonization of exposed regions to taxa from stream water.

The relatively low taxonomic resolving power of the 16S and 18S regions selected preclude conclusions about mechanisms of temporal or insect FS treatment heterogeneity in OTU abundance. Exceptions included *Pseudoalteromonas* which has been shown to affect larval aquatic insect settlement and development due to its strong tendency to form biofilm [118]. *Fluviicola* species are likely pathogens given taxonomic similarities to fish pathogens in the family Flavobacteriaceae [119]. Should insects selectively remove *Fluviicola*, benefits could accrue as individual develop microbiota on different tissues [32]. Eggs from predator treatments also experienced greater prevalence of *Saprolegnia*, an oomycete genus which includes a common fish pathogen [120, 121]. The effects of insect FS members themselves and collectively with members of stream microbial communities affected behavior and trait distributions of larval lake sturgeon in ways that can affect population levels of recruitment [22].

### Extensions of study findings to natural stream environments

Our experimental setting was artificial in the sense that replicated treatments only exposed sturgeon eggs to a single taxon within a feeding group. Natural stream ecosystems are inherently more complex [122, 123] in terms of structural and biotic features of stream substrate and alternative food sources available that could all lead to interactions among trophic levels that depart from experimental conditions simulated in this study. In natural streams, eggs are an ephemeral and novel resource that instar stages of aquatic insects may not be exposed to more than a single time. However, the taxa used in this study are highly abundant in the Black River [42], and thus could have significant lethal and/or non-lethal effects on incubating eggs. Using the Isonychiidae (collector-filter/facultative predator) as an example, we would expect taxa that express plasticity in feeding preference to be more opportunistic, including on episodically available egg resources. All experimental treatments purposely reduced complexity to understand variability associated with the reduced set of parameters. Future studies could benefit from inclusion of multiple predators and prey and higher structural environmental complexity. Given strong empirical evidence, as observed in this study, that predators can affect the behavior and phenotype of prey, such modifications can in turn affect other predators [124]. The presence of multiple insect taxa could also alter the outcome of interactions between insects and sturgeon eggs based on the combination of insect taxa and relative size/age.

## Supporting information

S1 Table Bacterial (16S) OTUs that contributed >5% of the variance used in the first discriminant function used to discriminate between insect treatments. OTU IDs are ranked in terms of total abundance of sequences across all samples. The taxonomic ID is the taxonomic assignment given by the SILVA database. Loadings are the proportion of the amount of variation contributed by the OTU to the total variation used in the discriminant function. The correlation coefficient is the strength of the relationship between the OTU abundance and the discriminant function, with positive correlations showing higher abundance in river water samples and negative correlations showing higher abundance in egg surface samples.
(DOCX)

S2 Table Bacterial (16S) OTUs that contributed >5% of the variance used in the second discriminant function used to discriminate between insect treatments. OTU IDs are ranked in terms of total abundance of sequences across all samples. The taxonomic ID is the taxonomic assignment given by the SILVA database. Loadings are the proportion of the amount of variation contributed by the OTU to the total variation used in the discriminant function. The correlation coefficient is the strength of the relationship between the OTU abundance and the discriminant function, with positive correlations showing higher abundance in Perlidae samples.
(DOCX)

S3 Table Bacterial (16S) OTUs that contributed >5% of the variance used in the discriminant function used to discriminate between sampling times. OTU IDs are ranked in terms of total abundance of sequences across all samples. The taxonomic ID is the taxonomic assignment given by the SILVA database. Loadings are the proportion of the amount of variation contributed by the OTU to the total variation used in the discriminant function. The correlation coefficient is the strength of the relationship between the OTU abundance and the discriminant function, with positive correlations showing higher abundance in T1 and negative correlations showing higher abundance during T2.
(DOCX)

S4 Table Lower eukaryotic OTUs that contributed >5% of the variance used in the second discriminant function used to discriminate between insect treatments. OTU IDs are ranked in terms of total abundance of sequences across all samples. The taxonomic ID is the taxonomic assignment given by the SILVA database. Loadings are the proportion of the amount of variation contributed by the OTU to the total variation used in the discriminant functions. The correlation coefficient is the strength of the relationship between the OTU abundance and the discriminant functions. Positive correlations with DF1 indicate that the OTU was more abundant in the T2 Control, Perlidae, and Isonychiidae treatments than other treatments. Positive correlations with DF2 indicated that the OTU was more abundant in Control treatments while negative correlations indicate the OTU was more abundant in Perlidae and Isonychiidae samples.
(DOCX)
