# Supplementary material for: Aquatic insects differentially affect lake sturgeon larval phenotypes and egg surface microbial communities
Source: PLoS One. 2022 Nov 21;17(11):e0277336. doi: 10.1371/journal.pone.0277336 (PMC9678266; doi:10.1371/journal.pone.0277336)
Supplement: S2 Table — OTU IDs are ranked in terms of total abundance of sequences across all samples. The taxonomic ID is the taxonomic assignment given by the SILVA database. Loadings are the proportion of the amount of variation contributed by the OTU to the total variation used in the discriminant function. The correlation coefficient is the strength of the relationship between the OTU abundance and the discriminant function, with positive correlations showing higher abundance in Perlidae samples. (DOCX) [file pone.0277336.s002.docx]

**S2 Table.** Bacterial (16S) OTUs that contributed >5% of the variance used in the second discriminant function used to discriminate between insect treatments. OTU IDs are ranked in terms of total abundance of sequences across all samples. The taxonomic ID is the taxonomic assignment given by the SILVA database. Loadings are the proportion of the amount of variation contributed by the OTU to the total variation used in the discriminant function. The correlation coefficient is the strength of the relationship between the OTU abundance and the discriminant function, with positive correlations showing higher abundance in Perlidae samples.

| ***OTU ID*** | **Taxonomic ID** | **Loadings** | **Correlation Coef** |
| --- | --- | --- | --- |
| *Otu0001* | Comamonadaceae | 0.158 | 0.245 |
| *Otu0019* | Enhydrobacter | 0.175 | 0.345 |
| *Otu0021* | Enterobacteriaceae | 0.151 | 0.391 |
| *Otu0086* | Psychroflexus | 0.07 | 0.433 |
