# Supplementary material for: Aquatic insects differentially affect lake sturgeon larval phenotypes and egg surface microbial communities
Source: PLoS One. 2022 Nov 21;17(11):e0277336. doi: 10.1371/journal.pone.0277336 (PMC9678266; doi:10.1371/journal.pone.0277336)
Supplement: S4 Table — OTU IDs are ranked in terms of total abundance of sequences across all samples. The taxonomic ID is the taxonomic assignment given by the SILVA database. Loadings are the proportion of the amount of variation contributed by the OTU to the total variation used in the discriminant functions. The correlation coefficient is the strength of the relationship between the OTU abundance and the discriminant functions. Positive correlations with DF1 indicate that the OTU was more abundant in the T2 Control, Perlidae, and Isonychiidae treatments than other treatments. Positive correlations with DF2 indicated that the OTU was more abundant in Control treatments while negative correlations indicate the OTU was more abundant in Perlidae and Isonychiidae samples. (DOCX) [file pone.0277336.s004.docx]

**S4 Table.** Lower eukaryotic OTUs that contributed >5% of the variance used in the second discriminant function used to discriminate between insect treatments. OTU IDs are ranked in terms of total abundance of sequences across all samples. The taxonomic ID is the taxonomic assignment given by the SILVA database. Loadings are the proportion of the amount of variation contributed by the OTU to the total variation used in the discriminant functions. The correlation coefficient is the strength of the relationship between the OTU abundance and the discriminant functions. Positive correlations with DF1 indicate that the OTU was more abundant in the T2 Control, Perlidae, and Isonychiidae treatments than other treatments. Positive correlations with DF2 indicated that the OTU was more abundant in Control treatments while negative correlations indicate the OTU was more abundant in Perlidae and Isonychiidae samples.

| ***OTU ID*** | **Taxonomic ID** | **DF1 Loadings** | **DF1 Corr** | **DF2 Loadings** | **DF2 Corr** |
| --- | --- | --- | --- | --- | --- |
| *Otu0004* | Saprolegnia | 0.051 | 0.544 | --- | --- |
| *Otu0010* | Saprolegnia | 0.200 | 0.462 | 0.053 | -0.340 |
| *Otu0038* | Unclassified | 0.410 | 0.496 | 0.240 | 0.464 |
| *Otu0057* | Peronosporomycetes | 0.262 | 0.369 | 0.596 | 0.538 |
